# Extraction of a Fragment of a Silver Catheter from the Bladder, by the Operation of Lithotomy

**Published:** 1860-06

**Authors:** 


*Extraction of a Fragment of a Silver Catheter from the Bladder, by the Operation of Lithotomy.*—Rev. Mr. J., while hastily performing on himself the operation of catheterism, which, on account of an old stricture, he is obliged daily to practice, broke the instrument within the urethra. As the catheter could not be felt in the course of the urethra, it was evident that the broken end had slipped into the bladder, and the lithotomy for its removal was immediately determined upon.

This was the third accident which had happened to the same patient. On the first occasion, I removed the fragment from the urethra by perineal section. At the second similar accident, two months ago, the patient complained for a few hours of severe pain, but which then entirely subsiding, it was believed the piece, instead of receding into the bladder, had escaped unnoticed from the urethra and fallen to the ground.

The catheters which were in such frequent use by the patient, had evidently become so attenuated by the oxidizing properties of the urine, that when roughly forced into the bladder, in the haste to relieve it, they were easily broken.

The operation performed for the extraction of the fragment was the lateral method, performed by cutting down on a staff in the usual manner. Through a small opening made in the prostate glands, forceps were introduced, and a body at once seized, which, on being drawn out, proved to be the piece of catheter lost in the bladder at the second accident, two months previously. It was blackened, and covered with dark, clotted blood. The piece which had been the object of the operation was with difficulty extracted, on account of the rough, broken end catching in the folds of the collapsed bladder. It was five inches in length.

The patient is now, six days after the operation, doing well, and I have ordered made for him a strong gold catheter, to insure him from another repetition of this dangerous accident.—*Dr. James Robarts, Carbondale, Ill., in Med. & Surg. Reporter.*

